# Involvement and Targeted Intervention of Mortalin-Regulated Proteome Phosphorylated-Modification in Hepatocellular Carcinoma

**DOI:** 10.3389/fonc.2021.687871

**Published:** 2021-07-29

**Authors:** Ye Yang, Ming Jin, Yi Dai, Wenqi Shan, Shuai Chen, Rong Cai, Haojun Yang, Liming Tang, Lei Li

**Affiliations:** ^1^Center for Global Health, School of Public Health, Nanjing Medical University, Nanjing, China; ^2^Department of General Surgery, The Affiliated Changzhou No. 2 Hospital of Nanjing Medical University, Changzhou, China

**Keywords:** hepatocellular carcinoma, angiogenesis and sorafenib resistance, mortalin, proteome phosphorylated-modification, targeted intervention

## Abstract

**Objectives:**

To reveal the mechanisms of the effects of mortalin in hepatocellular carcinoma (HCC) and to identify potential novel chemical inhibitors of mortalin.

**Materials and Methods:**

For the experiments, three HCC cell lines (HepG2 cells, Hep3B cells, and sorafenib-resistant HuH7 cells) and xenografted nude mice were used. For the clinical analysis, cohorts of 126 patients with HCC and 34 patients with advanced recurrent HCC receiving sorafenib therapy were examined.

**Results:**

Mortalin regulated the phosphorylation-modification of cancer-associated proteins and also regulated angiogenesis-related secretome to cause angiogenesis and sorafenib resistance in HCC cells. Two molecular mechanisms were identified. In one, *via* phosphatidylinositol 3-kinase (PI3K)/Akt signaling, mortalin regulated nuclear factor (NF)-κB and then activated vascular endothelial growth factor (VEGF)/vascular endothelial growth factor receptor (VEGFR)2 and granulocyte-macrophage colony-stimulating factor (GM-CSF), leading to neovascularization. In the other, mortalin regulated PI3K/Akt/β-catenin and then regulated Bcl-XL and Bcl-2, leading to the antiapoptosis effect of HCC. Treatment of the sorafenib-resistant xenografts with sorafenib in combination with mortalin knockdown facilitated the sorafenib-mediated inhibition of tumor growth and angiogenesis and increased apoptosis. Mortalin was a potential risk factor for HCC, predicting poor prognosis and sorafenib resistance. Finally, we showed that caffeic acid (C_9_H_8_O_4_) could bind to and induce the ubiquitination-mediated degradation of mortalin, which in turn blocked the abovementioned signaling pathways, leading to the inhibition of angiogenesis and the reversal of sorafenib resistance.

**Conclusions:**

Mortalin, which regulates the phosphorylation of cancer-associated proteins, caused angiogenesis and sorafenib resistance, and was a competitive risk factor for HCC. Caffeic acid can therefore be considered a novel chemical inhibitor that targets the action of mortalin and a potential treatment for HCC.

## Introduction

Hepatocellular carcinoma (HCC) is one of the most common solid tumors, and the fourth leading cause of cancer-related mortality worldwide (1). At present, the long-term outcome of patients with HCC is poor because of frequent recurrence and metastasis (2). Hematogenous metastasis represents a major shift in HCC biology: indeed, tumor angiogenesis, which plays a critical role in providing oxygen and nutrients, is necessary for HCC growth, intrahepatic and extrahepatic metastasis, and postsurgical recurrence (3). Thus, in addition to the curative therapies, resection, or transplantation (for the treatment of early-stage HCC, minority), antiangiogenesis strategies, such as sorafenib, have become the main approach for the treatment of advanced and recurrent HCC (which forms the majority of cases) (4). However, the therapeutic effects of these treatments are less than satisfactory. This is largely due to angiogenesis and the development of sorafenib resistance (5, 6). Consequently, the identification of novel therapeutic strategies for HCC is a priority.

Mortalin is a stress-inducible molecular chaperone that belongs to the family of heat shock proteins (7). The overexpression of mortalin is associated with increased malignancy, angiogenesis, and metastasis in HCC and early recurrence (8, 9). In the classical mechanism, mortalin sequestrates p53 in the cytoplasm, leading to the inactivation of p53 function and the suppression of apoptosis (10). However, unlike patients from Western countries, who have a low frequency (between 10% and 20%) of p53 mutations, patients with HCC from Southeast Asia (e.g., China and Japan) harbor a high frequency (up to 60%) of p53 mutations owing to chronic hepatitis B infection and aflatoxin B1 exposure (11, 12). Furthermore, the increased expression of mortalin is mainly closely associated with cell protection, which promotes the survival of cells under lethal conditions and acts as a “guardian” to combat stress and apoptosis (13). Collectively, in addition to the classical repression of p53 activity, the molecular mechanisms underlying the cancer-promoting effects of mortalin in HCC are very complicated.

In our present study, we focused on describing a p53-independent mechanism of mortalin that leads to angiogenesis and sorafenib resistance in HCC both *in vitro* and *in vivo*. Furthermore, we also identified the novel potential chemical inhibitors of mortalin *via* computer docking simulations and verified them using molecular biology techniques.

## Materials and Methods

### Cell Culture and Reagents

The HCC cell lines, HepG2 (wild-type p53), and Hep3B (p53 null) were obtained from the Institute of Biochemistry and Cell Biology, Shanghai Institutes for Biological Sciences, Chinese Academy of Sciences. The highly angiogenic and sorafenib-resistant HuH7 cell line (HuH7^SR^, with mutant p53) was established as described previously (14). The cells were cultured in Dulbecco’s Modified Eagle Medium (DMEM, Life Technologies/Gibco, Grand Island, NY), supplemented with 10% fetal bovine serum, 100 U/ml penicillin, and 100 μg/ml streptomycin (Gibco) in a 37°C humidified incubator with 5% CO_2_. A mycoplasma stain assay kit (Beyotime Co. Ltd., Haimen, China) was used for mycoplasma testing. Sorafenib (>99.89% purity) was purchased from Selleckchem Co. Ltd. (Houston, TX, USA), and 3,4-dihydroxycinnamic acid (caffeic acid, CaA, C_9_H_8_O_4_, ≥98.0% purity) and LY294002 [phosphatidylinositol 3-kinase (PI3K) inhibitor, ≥98.0% purity] were purchased from Sigma-Aldrich (Shanghai, China). All other reagents used in the present study were of analytical grade or the highest grade available.

### Xenografts in Nude Mice and Immunohistochemistry

The *in vivo* protocols were approved by the Nanjing Medical University Institutional Animal Care and Use Committee (2017-KY010). BALB/c nude mice were obtained from the SLRC Laboratory Animal Center (Shanghai, China) and were usually kept in a specific pathogen-free and temperature-controlled environment (20°C–22°C) under a 12-h light–dark cycle, with free access to drinking water and chow, as described previously (14). To establish the xenograft study, 2 × 10^6^ cells in 100 μl of Matrigel were injected subcutaneously into the right armpit of each mouse, and mice were fed normally for 3 weeks. The dose of sorafenib (Selleckchem, Houston, TX, USA) administered was 60 mg/kg body weight (BW) *via* gavage, with negative control (NC)-siRNA or mortalin-targeted siRNA (100 nM, Santa Cruz Biotechnology, CA, USA; **Supplementary Table S1**) administered *via* intratumoral injection every 3 days. The tumor volume was calculated using the formula: V = ½ (width^2^ × length). After 18 days, the mice were killed, and the tumor tissues were removed for further investigation. For immunohistochemistry (IHC), the sections were mounted on silanized slides, dewaxed in xylene, dehydrated in ethanol, boiled in 0.01 M citrate buffer (pH 6.0) for 20 min in a microwave oven, and then incubated with 3% hydrogen peroxide for 5 min. The sections were washed in PBS, incubated in 10% normal bovine serum albumin for 5 min, and then incubated with the primary antibody at 4°C overnight. The slides were then incubated with a horseradish peroxidase-conjugated secondary antibody at room temperature for 30 min. The samples were then visualized using diaminobenzidine, dehydrated, cleared, mounted, and photographed using a panoramic-scan digital slice scanning system (3DHISTECH Co. Ltd., Budapest, Hungary). The graphs were analyzed using Image-Pro Plus 6.0 software, as described previously (14, 15). The criteria determining the immunostaining score are listed in **Supplementary Table S2**.

### Patients and Tissue Microarray

This study was approved by the Medical Ethics Committee of the Affiliated Changzhou No. 2 Hospital of Nanjing Medical University, and written informed consent was obtained from each patient for the study of tissue excised from the surgical specimens. The clinical pathology data are listed in **Supplementary Tables S3** and **S4**. The tissue microarray was constructed by Shanghai Zhuoli Biotechnology Co. Ltd. (Zhuoli Biotechnology Co., Shanghai, China). In each case, 1–2-μm-thick sections were cut from paraffin tissue blocks, dewaxed, pretreated, and placed on glass slides using an adhesive tape transfer system in order to avoid ultraviolet cross-linking. All reactions were performed using an automated staining device (Roche, Ventana Medical Systems, Oro Valley, AZ, USA). The immunostaining was quantified by two independent researchers who were blinded to the patient details described above.

### Phosphoprotein-Specific Microarray Analysis

The phosphoprotein profiling was designed and manufactured using the phospho-explorer antibody array (PEX100) from Wayen Biotechnology (Shanghai, China). The samples were processed by protein extraction, lysate and marker buffer replacement, protein quantification, biotin labeling, and other operations according to the manufacturer’s recommendations. Then, array closure, incubation, protein and array hybridization, and streptavidin combined with Cy3 detection were performed. The Surescan Dx Microarray Scanner was used to scan the array and obtain the data. The microarray contains 1,318 antibodies; each phosphorylation site was detected by two different types of antibodies to identify the phosphorylation status. Two replicates of each antibody were present and were located in two symmetrical blocks. The data for each phosphorylation site are presented as the mean of two biological replicates located in the two symmetrical blocks, partly narrowed the margin of error, with data correction performed at the same time. GenePix Pro 6.0 software was used to read the original data, including the fluorescence signal and background. A ratio was used to compute the extent of protein phosphorylation from the equation: Phosphorylation ratio = Phosphorylated value/unphosphorylated value. The data were shown in **Supplementary Datasheet S1**.

### Angiogenic Antibody Array

A Quantibody^®^ Human Angiogenesis Array manufactured by RayBiotech (Guangzhou, China) was used to detect angiogenic factors in accordance with the manufacturer’s instructions. Briefly, freshly frozen samples that were stored at -80°C were thawed and dissolved in lysis buffer to disintegrate the protein, and the protein concentrations were measured. The glass chip was air-dried at room temperature for 1–2 h, and standard dilutions of cytokine solutions were prepared. Then, 100 μl of sample diluent was added into each well on the glass chip and incubated at room temperature for 30 min to block nonspecific binding to the slides. The buffer was decanted from each well, and 100 μl of standard cytokines or sample was added to each well; then, the arrays were incubated at room temperature for 1–2 h. The glass chip was washed using Thermo Scientific Wellwash Versa Chip washer, incubated with the antibody detection cocktail and Cy3 equivalent dye-Streptavidin. Finally, an InnoScan 300 Microarray Scanner (wavelength, 532 nm; resolution, 10 µm) was used to measure the fluorescence, GenePix Pro 6.0 software was used to extract the data, and Quantibody^®^ Q-Analyzer software was used to analyze the data. The data for each of the angiogenic factors were presented as the mean of two biological replicates located in two symmetrical blocks, and the data were shown in **Supplementary Datasheet S2**.

### Analysis of Biological Functions

The STRING database (Search Tool for the Retrieval of Interacting Genes; https://string-db.org) was employed to construct the protein–protein interaction network. The DAVID database (Database for Annotation, Visualization and Integrated Discovery; http://david.ncifcrf.gov) was employed to process the Gene Ontology (GO) and Kyoto Encyclopedia of Genes and Genomes (KEGG) pathway analyses. A p-value of <0.05 was set as the cutoff criterion for significant enrichment.

### Determination of the Binding Status of Mortalin With Caffeic Acid or Ubiquitin

Huh7^sr^ Cells were pretreated with 0 or 20 μM MG-132 for 2 h and then exposed to 20 μM CaA for 6 h. Then, the cells were extracted with immunoprecipitation (IP) lysis buffer (Beyotime) for 30 min. The preparations were centrifuged, and 100 μg of total protein was incubated with anti-mortalin antibody at 4°C overnight. The protein–antibody complexes were incubated with IgG Sepharose beads (Beyotime) at 4°C for another 12 h. Subsequently, the supernatants were removed (positive control), and the beads were washed three times (the residual supernatants served as a negative control), boiled, and centrifuged. Then, the test substance was dissolved in water and analyzed by high-performance liquid chromatography–mass spectrometry (HPLC-MS) using the Thermo Fisher Q Exactive Plus LC-MS system with the CaA standard as the positive control. To determine the extent of protein ubiquitination, a co-immunoprecipitation assay was performed as described previously (16). Briefly, HuH7^SR^ cells were treated and extracted, and the IP complex was prepared as described above. Then, the samples were analyzed by performing a Western blotting assay with ubiquitin antibody.

### Functional Assays

The other functional assays are presented in the *Supplementary Materials and Methods* section.

### Statistical Analysis

The datasets were compared using GraphPad 8.0 (GraphPad Software, Inc., CA, USA), and the data were presented as mean ± SD. The differences were analyzed using Student’s *t*-test, one-way analysis of variance followed by Dunnett’s *t*-test, or two-way analysis of variance followed by Sidak’s multiple comparison test, or linear regression. Survival curves were estimated using the Kaplan–Meier method, and differences in the survival distributions were evaluated by the log-rank test. A p-value of <0.05 was considered statistically significant.

## Results

### Mortalin Caused Angiogenesis and Sorafenib Resistance in Hepatocellular Carcinoma Cells

**Figure 1:**
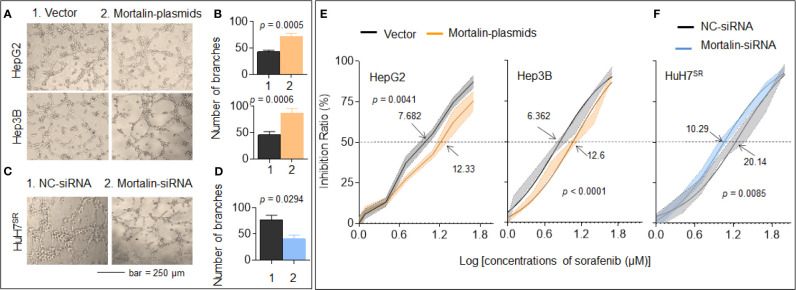
Mortalin causes angiogenesis and sorafenib resistance in hepatocellular carcinoma (HCC) cells. HepG2 and Hep3B cells were transfected with mortalin-plasmids, and HuH7^SR^ cells were transfected with mortalin-siRNA. **(A, C)** Capillary tube formation assay of the effects of mortalin on tube formation in human umbilical vein endothelial cells (HUVECs). **(B, D)** Quantitative analysis, determined in triplicate, of tube formation. The differences were analyzed by Student’s *t*-test. **(E, F)** After the three HCC cell types were transfected with mortalin-siRNA or mortalin-plasmids, they were treated with different concentrations of sorafenib for 24 h. The cell viability was determined in triplicate, and IC_50_ values were calculated. The differences were analyzed using two-way analysis of variance followed by Sidak’s multiple comparison test.

First, we found that mortalin expression in HuH7^SR^ cells was significantly higher than that in HepG2 and Hep3B cells (**Supplementary Figure S1**). Then, the mortalin-plasmids were transfected to HepG2 and Hep3B cells, while the mortalin-siRNAs were transfected to HuH7^SR^ cells. We next evaluated the effects of mortalin on angiogenesis in the three cell lines and found that the tube formation ability of mortalin-plasmid-transfected cells were significantly increased, whereas knockdown of mortalin resulted in the opposite effect (**Figures 1A–D** and **Supplementary Figure S1**). Next, we investigated the effects of mortalin on sorafenib resistance. Similarly, the knockdown of mortalin increased the efficiency of sorafenib, whereas the overexpression of mortalin induced the opposite effect. The IC_50_s of sorafenib in mortalin-plasmid- or mortalin-siRNA-transfected cells with respective control cells were as follows: 12.33 vs. 7.682 for HepG2 cells, 12.6 vs. 6.362 for Hep3B cells, and 10.29 vs. 20.14 for HuH7^SR^ cells (**Figures 1E, F** **)**. Collectively, these results indicated that mortalin may be an effective factor that could enhance angiogenesis and contribute to sorafenib resistance in HCC cells. Moreover, mortalin may cause the abovementioned effect in both a p53-dependent and a p53-independent manner.

### Mortalin Regulated Phosphorylation in Cancer-Associated Proteins

**Figure 2:**
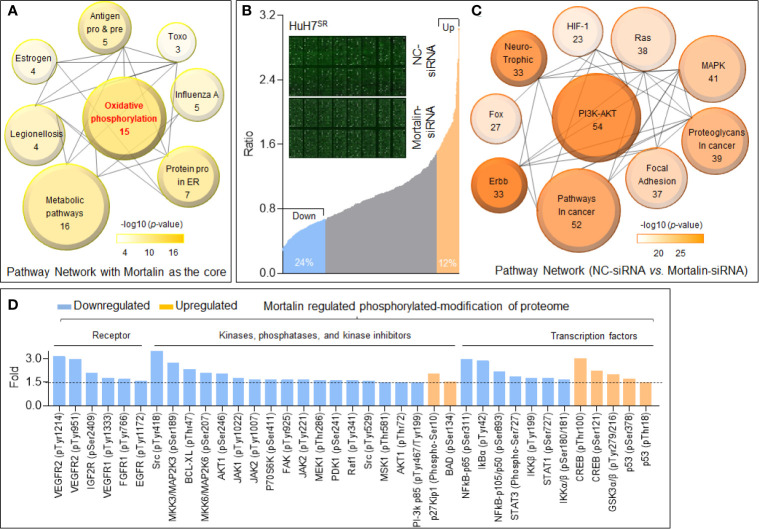
Mortalin regulates the phosphorylation of cancer-associated proteins in hepatocellular carcinoma (HCC) cells. **(A)** Kyoto Encyclopedia of Genes and Genomes (KEGG) pathway enrichment analyses of the 20 most frequently altered neighbor interactors and 30 indirect interactors with mortalin from STRING database (Search Tool for the Retrieval of Interacting Genes). The size of the circle indicates the number of genes enriched in the item, and different color shades indicate the size of p-value. **(B)** Phospho-antibody microarray analysis of the changes in expression of phosphoproteins following mortalin knockdown in HuH7^SR^ cells. Upregulation of phosphorylation by more than 50% (1.5-fold) is shown in orange, and downregulation of more than 33% (1.5-fold) is shown in blue. **(C)** KEGG pathway enrichment analyses of the proteins with upregulation or downregulation of phosphorylation of more than 1.5-fold upon mortalin knockdown; the top 10 pathways are shown. **(D)** Phospho-antibody microarray data showing the phosphorylation levels of critical receptors, kinases, phosphatases, kinase inhibitors, and transcription factors that are involved in the top 5 pathways listed in panel **(C)** The data presented for each phosphorylation site are the mean of two biological replicates.

In addition to the classic role of mortalin in the inactivation of p53, it also improves cancer cell survival and enhances tumor progression (especially in drug-resistant tumors) through other methods, such as synergistic effects with human telomerase reverse transcriptase and heterogeneous nuclear ribonucleoprotein K (17). First, we employed the STRING database to predict the potential mortalin-regulated downstream signal transduction mechanisms. With mortalin employed as the central molecule, a protein-protein interaction network was generated for the 20 most frequently altered neighbor interactors and the 30 surrounding indirect interactors (**Supplementary Figure S2**). Next, we employed the DAVID database to conduct KEGG pathway analysis on the 50 factors identified. The enrichment analysis identified eight pathways related to the functions of mortalin, and the highest ranked function was oxidative phosphorylation (**Figure 2A**). Previous studies have shown that mortalin was involved in tumor development through its impact on the phosphorylation of several key molecules (18, 19). Therefore, to clarify the effects of mortalin on protein phosphorylation, control and mortalin knockdown HuH7^SR^ cells were analyzed using a phospho-antibody microarray. We identified a spectrum of proteins with a change of more than 1.5-fold (i.e., a 50% increase or a 33% decrease) in phosphorylation compared with the mock-transfected control (**Figure 2B**). In the KEGG enrichment analysis of the array, the PI3K/Akt pathway was ranked most highly (**Figure 2C** and **Supplementary Figure S3**). Furthermore, many of these phosphorylated proteins are vital to tumor progression, and the knockdown of mortalin resulted in the extensive suppression of protein phosphorylation. As shown in **Figure 2D**, the phosphorylation of vascular endothelial growth factor receptor (VEGFR), insulin-like growth factor receptor (IGFR), fibroblast growth factor receptor (FGFR), and epidermal growth factor receptor (EGFR), which are receptors with extracellular signal transduction functions, is clearly declined. The results also revealed a reduction in the phosphorylation of many key components crucial to signaling pathways, such as PI3K/Akt (PI3K-p85, AKT1), mitogen-activated protein kinase (MAPK) (MEK1, MKK3/6), NF-κB (IKKβ, IκBα, p65, p105/50), and Janus kinase–signal transducer and activator of transcription (JAK-STAT) (JAK1/2, STAT1). Meanwhile, several factors involved in the negative regulation exhibited the opposite trend. Collectively, these results indicated that mortalin could regulate the phosphorylation of cancer-associated proteins, and that PI3K/Akt might be a key downstream pathway, regulated by mortalin, in highly angiogenic and sorafenib-resistant HCC cells.

### Identification of PI3K/Akt as an Important Downstream Factor of Mortalin, Regulating NF-Kb and GSK3/B-Catenin in Hepatocellular Carcinoma Cells

**Figure 3:**
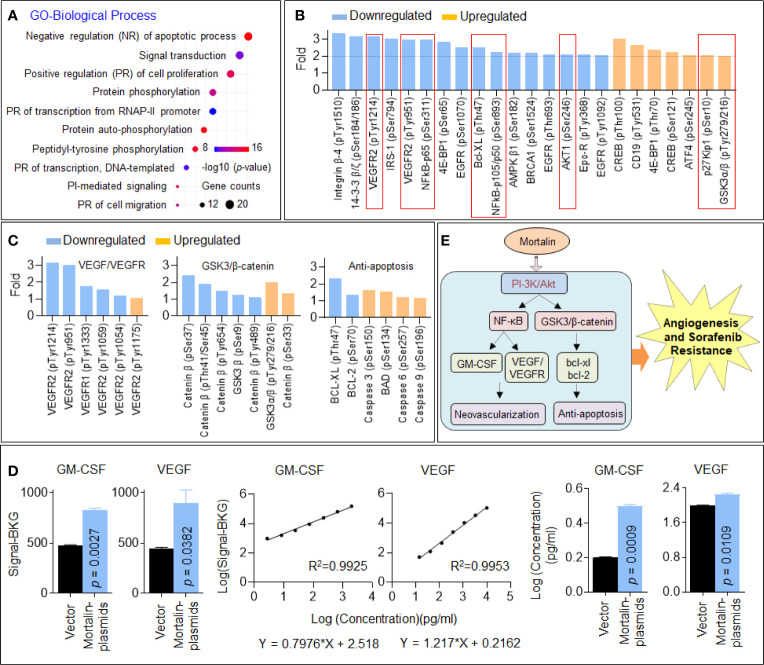
Identification of phosphatidylinositol 3-kinase (PI3K)/Akt as an important downstream factor regulating vascular endothelial growth factor (VEGF) and glycogen synthase kinase (GSK)3/β-catenin in hepatocellular carcinoma (HCC) cells. **(A)** Gene Ontology (GO) biological process analysis of the 54 proteins enriched in the PI3K/Akt pathway. **(B)** The proteins in the PI3K/Akt signaling pathway with the most significant changes in phosphorylation (greater than 2-fold). **(C)** The changes in the phosphorylation of proteins involved in VEGF/vascular endothelial growth factor receptor (VEGFR) signaling, GSK3/β-catenin signaling, and apoptosis. The data of each phosphorylation site level exhibited were the mean of two biological replicates. **(D)** The fluorescence signal values, standard curves, and calculated concentrations of granulocyte-macrophage colony-stimulating factor (GM-CSF) and VEGF in mortalin-plasmid- and vector-transfected HepG2 cells in the angiogenic antibody array. Means and standard deviations were calculated from replicate samples, and the differences were analyzed using Student’s *t*-test. **(E)** An outline map speculating the potential mechanisms involved in mortalin-induced angiogenesis and sorafenib resistance.

We further investigated the underlying mechanisms using GO analysis based on the proteins enriched in the PI3K/Akt signaling pathway. As shown in **Figure 3A**, the top-ranked biological process was negative regulation of apoptotic process. Next, we analyzed the phosphoproteins with the most significant changes in the PI3K/Akt signaling pathway and listed those downregulated or upregulated at least 2-fold compared with the control HuH7^SR^ cells. Here, in mortalin knockdown cells, the phosphorylation of many important factors including AKT1, VEGFR2, NF-κB, and Bcl-XL was significantly decreased, whereas the phosphorylation of p27kiP1 and glycogen synthase kinase (GSK)3β was significantly increased (**Figure 3B**). Then, we further investigated the differential phosphorylation of proteins enriched in vascular endothelial growth factor (VEGF)/VEGFR, GSK3/β-catenin, and antiapoptosis signaling and confirmed that the abovementioned three signaling pathways were all inactivated in mortalin knockdown cells (**Figure 3C**). Previous studies revealed that VEGFR2 would be targeted by sorafenib; therefore, to sensitize cells to sorafenib, another pathway (VEGF-independent manner) might be involved (20, 21). Therefore, it is important to analyze the angiogenic proteome in HCC cells. Here, we employed an angiogenesis array to detect angiogenic factors in the HCC cells in mortalin-overexpressing HepG2 cells. The results showed that, among all the factors that could be detected, the levels of granulocyte-macrophage colony-stimulating factor (GM-CSF) and VEGF were markedly increased by the transfection of mortalin-plasmids (**Supplementary Datasheet S2** and **Figure 3D**). Moreover, NF-κB, which is regulated by the PI3K/Akt signaling pathway, was associated with VEGF expression. In addition, NF-κB activity and VEGF expression were also correlated with microvessel density in tissues and cell lines during *in vitro* angiogenesis, confirming NF-κB as one of the critical upstream regulators of VEGF (22, 23). Meanwhile, GM-CSF is also one of the classic factors regulating angiogenesis that is regulated by NF-κB signaling (24, 25). The evidence indicated that the activation of GM-CSF *via* NF-κB might be another way that is regulated by mortalin of HCC to promote angiogenesis besides VEGF signaling. Based on these results, we proposed two potential methods. In one, PI3K/Akt regulated VEGF/VEGFR and GM-CSF *via* NF-κB signaling, leading to neovascularization; in the other, PI3K/Akt regulated GSK3/β-catenin, leading to an antiapoptosis effect. The two main neoplastic biological processes described are both involved in mortalin-induced angiogenesis and sorafenib resistance in HCC (**Figure 3E**).

### Verification of Microarray and Bioinformatics Results *In Vitro* and *In Vivo*

**Figure 4:**
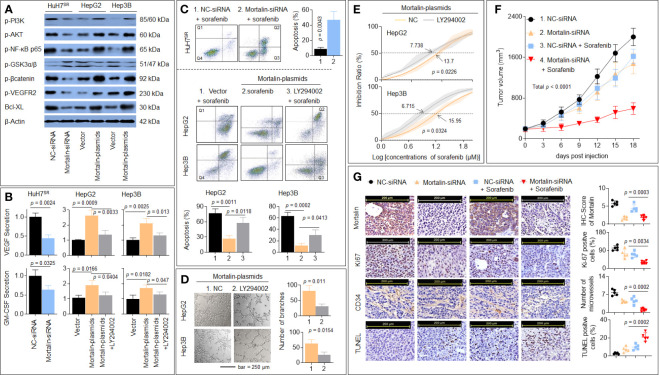
Verification of microarray and bioinformatics results *in vitro* and *in vivo*. **(A)** HuH7^SR^ cells were transfected with mortalin-siRNA, and HepG2 and Hep3B cells were transfected with mortalin-plasmids. Western blotting assay and quantitative analyses of the expression of phosphatidylinositol 3-kinase (PI3K)-p85 (Tyr458), Akt (Ser473), nuclear factor (NF)-κB p65 (Ser536), vascular endothelial growth factor receptor (VEGFR)2 (Tyr951), glycogen synthase kinase (GSK)3β (Tyr216), β-catenin (Thr41/Ser 45), and Bcl-XL. **(B)** HuH7^SR^ cells were transfected with mortalin-siRNA, and HepG2 and Hep3B cells were transfected with mortalin-plasmids, pretreated with LY294002, and the medium was collected. Triplicate ELISA analyses of VEGF and granulocyte-macrophage colony-stimulating factor (GM-CSF) protein secretion. **(C)** After transfection, these cells were treated with 10 μM sorafenib. Flow cytometric analyses of the percentage of cells in apoptosis (Q1: necrotic cells, Q2: late apoptosis, Q3: early apoptosis, Q4: living cells). From the histograms, the proportion of cells in total apoptosis (Q2 + Q3) was statistically analyzed. **(D)** Capillary tube formation assay analyses of mortalin-plasmid-transfected HepG2 and Hep3B cells, in the absence of LY294002, on tube formation in human umbilical vein endothelial cells (HUVECs); a quantitative analysis is shown. **(E)** Mortalin-plasmid-transfected HepG2 and Hep3B cells were treated with different concentrations of sorafenib for 24 h in the absence of LY294002. The cell viability was determined in triplicate, and the IC_50_ values were calculated. **(F, G)** The HuH7^SR^ cell xenografts were treated with mortalin-siRNA alone, sorafenib alone, or sorafenib plus mortalin-siRNA. **(F)** The volumes of xenografted tumors. **(G)** Immunohistochemistry (IHC) staining of the mortalin, ki67, CD34, and terminal deoxynucleotidyl transferase biotin-dUTP nick end labeling (TUNEL) staining. The differences were analyzed using Student’s *t*-test and one-way analysis of variance followed by Dunnett’s *t*-test.

To verify the abovementioned hypothesis, we first determined the regulatory effect of mortalin on the PI3K/Akt, VEGF/VEGFR, and β-catenin/anti-apoptosis pathways, as determined by the level of phosphorylation of PI3K-p85 (Tyr458), Akt (Ser473), NF-κB p65 (Ser536), VEGFR2 (Tyr951), GSK3β (Tyr216), β-catenin (Thr41/Ser 45), Bcl-XL, and the extent of apoptosis. As shown in **Figure 4A**, the knockdown of mortalin decreased the phosphorylation (activation) of PI3K/Akt/NF-κB/VEGFR2 but increased the phosphorylation of GSK3β, which in turn inactivated the β-catenin/Bcl-XL pathway. The knockdown of mortalin in HuH7^SR^ cells attenuated the secretion of VEGF and GM-CSF, whereas the overexpression of mortalin in HepG2 and Hep3B cells increased the secretion; however, the inhibition of PI3K/Akt led to the opposite effects (**Figure 4B**). In addition, the knockdown of mortalin enhanced apoptosis, but the opposing trend was observed when mortalin was overexpressed, although apoptosis was significantly elevated when the cells were pretreated with LY294002 (**Figure 4C**). LY294002 was only used as a pretreatment; it was removed after treatment and not present in the conditioning medium. Here, compared with the mortalin-plasmid-transfected cells, the tube formation ability of the LY294002-pretreated cells was markedly reduced (**Figure 4D**). Furthermore, the IC_50_ of sorafenib was also decreased in both HepG2 and Hep3B cells subjected to the LY294002 pretreatment (**Figure 4E**). We also performed *in vivo* experiments to confirm our results. As shown in **Figures 4F, G**, treatment of the xenografts with sorafenib alone or knockdown of mortalin inhibited tumor growth and angiogenesis. In combination, the knockdown of mortalin facilitated the sorafenib-mediated inhibition of tumor growth and angiogenesis and subsequently enhanced apoptosis. Collectively, these results suggested that the knockdown of mortalin blocked the PI3K/Akt pathway, which in turn inactivated the VEGF/VEGFR, GM-CSF, and β-catenin/antiapoptosis signaling pathways, leading to the attenuation of angiogenesis and sorafenib resistance in HCC cells.

### The Clinical Significance of Mortalin in Hepatocellular Carcinoma

**Figure 5:**
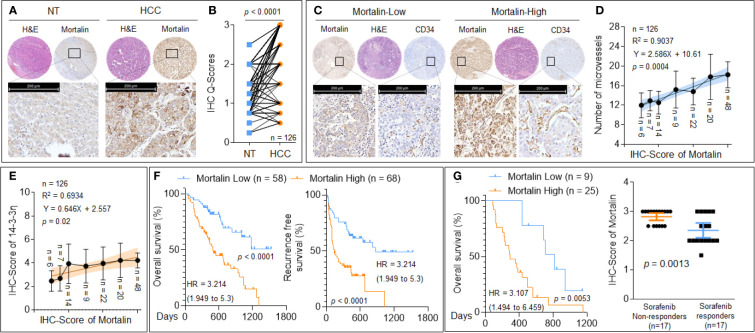
The clinical significance of mortalin in hepatocellular carcinoma (HCC). **(A, B)** Tissue microarray and immunohistochemistry (IHC) analyses of mortalin in samples from patients with HCC compared with adjacent non-tumor liver tissues. **(C)** Tissue microarray and IHC analyses of mortalin and CD34 in HCC samples. **(D)** Relationship between the expression of mortalin and the number of microvessels. **(E)** Triplicate tissue microarray and IHC analyses of mortalin and 14-3-3η in HCC sample; their relationship is exhibited. **(F)** Kaplan–Meier analyses of the prognostic significance of mortalin in 126 patients with HCC. (**G**, left) Kaplan–Meier analyses of the prognostic significance of mortalin in 34 patients with advanced recurrent HCC. (**G**, right) Analyses of the mortalin levels in sorafenib nonresponders and sorafenib responders. IHC staining was performed in triplicate. The differences were analyzed using Student’s *t*-test and linear regression.

We then evaluated the expression of mortalin in patients with HCC. As shown in **Figures 5A, B**, compared with the adjacent non-tumor liver tissues, a considerable increase in mortalin expression was observed in HCC tissues. To investigate the association between mortalin expression and angiogenesis, the HCC specimens were divided into “mortalin-low” vs. “mortalin-high” groups according to the IHC-Q-Scores. The number of microvessels in the specimens in the mortalin-high group was significantly higher than those in the mortalin-low group (**Figure 5C**). Moreover, a significant positive correlation was found between mortalin expression and the number of microvessels (**Figure 5D**). We also found that there was a positive correlation between mortalin and 14-3-3η [an inducer of angiogenesis and sorafenib resistance described in our previous study (14, 15)] (**Figure 5E**). Finally, Kaplan–Meier survival analysis showed that patients in the mortalin-high group had poorer overall survival and recurrence-free survival than those in the mortalin-low group (**Figure 5F**). The survival analysis of 34 patients with advanced recurrent HCC (who had received combined sorafenib treatment and transarterial chemoembolization therapy) revealed the same trend (**Figure 5G**, left). In addition, we further defined the median survival of the cohort, 434 days, as the criterion for response to sorafenib: patients who survived less than 434 days were defined as sorafenib nonresponders, whereas patients who survived longer than 434 days were defined as sorafenib responders. There was a significant difference between the sorafenib responders and nonresponders (**Figure 5G**, right). These results indicated that mortalin may be regarded as a potential risk factor for HCC, predicting poor prognosis and sorafenib resistance.

### Identification of a Novel Potential Chemical Inhibitor of Mortalin: Caffeic Acid

**Figure 6:**
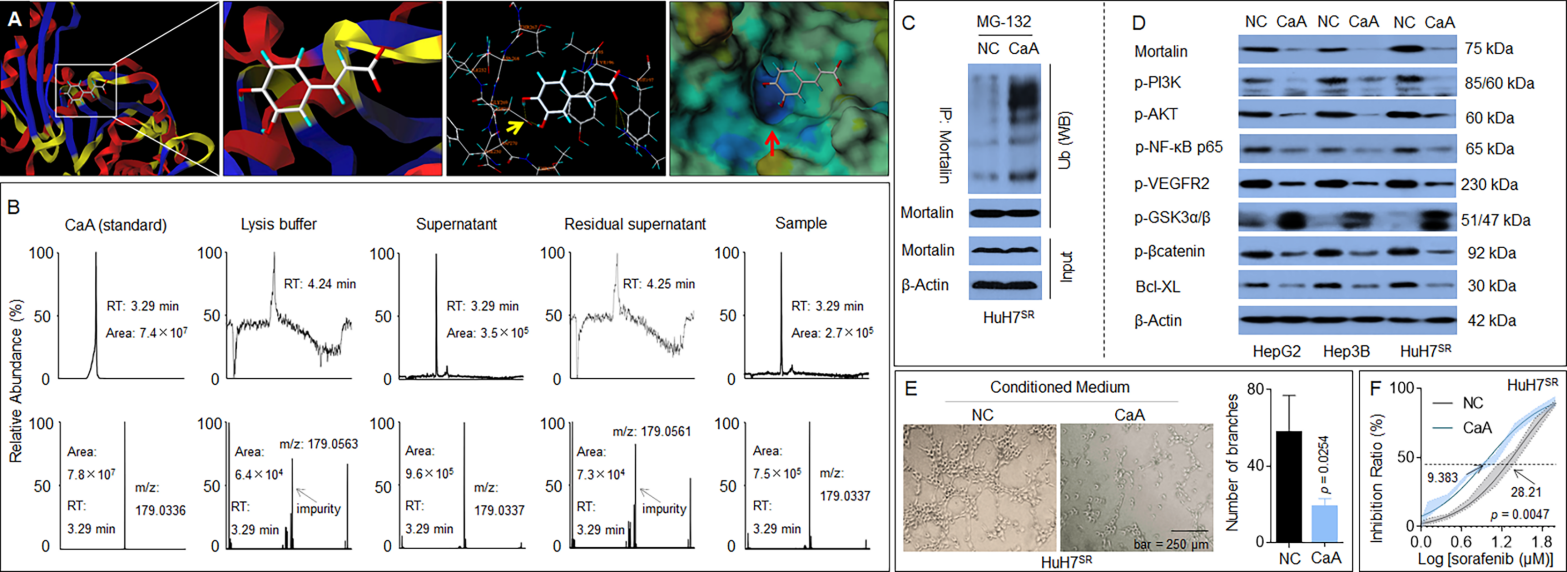
Identification of a novel potential chemical inhibitor for mortalin: Caffeic acid (CaA). **(A)** SYBYL-X software was used to analyze the binding of CaA to mortalin. After HuH7^SR^ cells were pretreated with 20 μM MG-132 for 2 h, they were exposed to 20 μM CaA for 6 h. Mortalin was immunoprecipitated using a specific antibody. **(B)** Liquid chromatography–mass spectrometry (LC-MS) quantification of the content of CaA in the mortalin-immunoprecipitation complex. **(C)** Western blotting analysis of the expression of ubiquitin in the mortalin-immunoprecipitation complex. **(D)** HepG2, Hep3B, and HuH7^SR^ cells were treated with 20 μM CaA for 24 h. Western blotting analysis of the expression of mortalin, phosphatidylinositol 3-kinase (PI3K)-p85 (Tyr458), Akt (Ser473), nuclear factor (NF)-κB (Ser536), vascular endothelial growth factor receptor (VEGFR)2 (Tyr951), glycogen synthase kinase (GSK)3β (Tyr216), β-catenin (Thr41/Ser 45), and Bcl-XL. **(E)** Capillary tube formation assay of the effects of CaA on tube formation in human umbilical vein endothelial cells (HUVECs); a quantitative analysis, determined in triplicate, is provided. **(F)** HuH7^SR^ cells were treated with 20 μM CaA and then treated with different concentrations of sorafenib for 24 h. The cell viability was determined in triplicate, and the IC50 values were calculated. The differences were analyzed using Student’s *t*-test and two-way analysis of variance followed by Sidak’s multiple comparison test.

MKT-077 (C_21_H_22_ClN_3_OS_2_) is a classic chemical inhibitor of mortalin, which functions by eliminating mortalin–p53 interactions but does not alter the expression of mortalin (26). Previous studies revealed that caffeic acid phenethyl ester (CAPE; C_17_H_16_O_4_), a specific inhibitor of NF-κB activation, was also able to induce the disruption of the mortalin–p53 complex, leading to nuclear translocation and the activation of p53, and was accompanied by a decrease in the expression of mortalin *via* transcriptional inactivation (27). However, the effects of these chemicals on the stability of the mortalin protein remain unclear. Our previous study revealed that, similar to CAPE, CaA was also an effective NF-κB inhibitor (28), and that NF-κB was an upstream transcriptional regulator of mortalin (29). Moreover, *via* computational docking studies using SYBYL-X software, we found that the basic skeleton structure of CAPE, CaA (C_9_H_8_O_4_), could bind to mortalin, and that the binding region of CaA to mortalin was the same as that of MKT-077 (**Figure 6A**). Thus, we adopted an innovative approach, combining both IP and liquid chromatography–mass spectrometry (LC-MS) techniques, to reveal the interaction between CaA and mortalin. Mortalin was immunoprecipitated by a specific antibody, and its CaA-binding status was determined by LC-MS to investigate if the IP complex contained CaA. As shown in **Figure 6B**, the retention time of CaA was 3.29 min, and the area of CaA in the positive control group (total protein, supernatant) was 9.6 × 10^5^. The area of CaA in the immunoprecipitated complex (experimental group, sample) was 7.5 × 10^5^. CaA was almost undetectable in the negative control groups (lysis buffer and residual supernatant). Moreover, CaA treatment increased the ubiquitination of mortalin in HuH7^SR^ cells (**Figure 6C**), decreased the expression/phosphorylation of mortalin, PI3K-p85 (Tyr458), Akt (Ser473), NF-κB(Ser536), VEGFR2 (Tyr951), β-catenin (Thr41/Ser 45), and Bcl-XL (**Figure 6D**). We also used a previously established MHCC97H xenograft model and applied CaA (30, 31) to confirm the effects of CaA on the expression/phosphorylation of the abovementioned factors *in vivo* and found that CaA treatment inhibited mortalin, PI3K/Akt, NF-κB, VEGFR2, and β-catenin/Bcl-XL (**Supplementary Figure S4**). Moreover, when we treated HuH7^SR^ cells with CaA, the tube formation ability and the IC_50_ of sorafenib were lower than those in the control group (**Figures 6E, F** **)**. Collectively, these results indicated that CaA inhibited the activity of mortalin by both transcriptional (indirectly, *via* blocking NF-κB) and posttranscriptional (directly, *via* targeting and inducing the ubiquitin-mediated degradation of mortalin) modifications in HCC cells.

## Discussion

Mortalin is an abundant mitochondrial protein also found in the mitochondria, endoplasmic reticulum, plasma membrane, and cytoplasmic vesicles. It participates in various biological processes, including proliferation, functional maintenance, and stress response in cancer cells and promotes tumor progression (7, 32). Studies have also revealed that mortalin has an antiapoptotic effect (13). The loss of mortalin induces mitochondrial dysfunction and is always accompanied by a decrease in ATP and an increase in reactive oxygen species (ROS) generation (33). The abnormal phosphorylation of several molecules and the regulation of a series of key downstream signal molecules increase the antioxidant capacity of tumor cells, which are also essential requirements for cells to obtain and maintain the redox balance. In the subset of drug-resistant tumor cells, two biological behaviors (disorder of the redox balance and abnormal phosphorylation) occur in parallel; it is inevitable that these behaviors will mutually coexist and have a synergic effect. There is no inconsistency between oxidative phosphorylation and the classical role of mortalin in the regulation of ROS generation. However, the regulatory role of mortalin in protein phosphorylation remains unconfirmed. Here, we found that mortalin caused angiogenesis and sorafenib resistance *via* the regulation of phosphorylated cancer-associated proteins in HCC. The 14-3-3η is an isoform of the 14-3-3 family of proteins, which are phosphoserine/threonine-binding proteins (34). We previously showed that the 14-3-3η isoform was a novel neoplastic factor of HCC, inducing growth, angiogenesis, and multidrug resistance (MDR) properties (14, 15). Here, a positive correlation between the expression of mortalin and 14-3-3η was found, suggesting that the mortalin-induced phosphorylation of the cancer-associated proteins may be mediated by 14-3-3η. This hypothesis requires further confirmation.

Mechanically, PI3K/Akt is a key signaling pathway of particular relevance in cancer progression, and the hyperphosphorylation of PI3K and the activation of Akt are considered crucial factors that contribute to perturbation of the antiapoptotic, stability, and normal functions (35). The enhancement of signal transduction by the PI3K/Akt pathway is a key contributor to neovascularization (36). Currently, VEGF has been considered the most potential angiogenesis-inducing factor. In the VEGF/VEGFR pathway, VEGFR2 is the most important VEGFR. In our study, we found that VEGFR2 was remarkably downregulated, including at several classic phosphorylation sites, such as Tyr951 and Tyr1059, which may promote the neovascularization and regeneration of tumor cells, contributing to the development of cancer (37, 38). In addition, GSK3 functions as an important downstream regulatory switch for numerous signaling pathways involved in the formation of tumors, including the regulation of the Wnt/β-catenin pathway (39, 40). In the pathway, activated AKT could bind to the GSK3β complex and then phosphorylate GSK3β at Ser9 and increase the β-catenin levels (41, 42) but inhibit GSK3β phosphorylation at Tyr216, also causing the activation of β-catenin (43). Here, we found that the phosphorylation of GSK3β at Ser9 was downregulated, whereas the phosphorylation of GSK3β at Tyr216 was upregulated in mortalin knockdown cells, which inhibited the phosphorylation of β-catenin at several main sites, such as Ser37, Thr41, and Tyr654. This downregulation of phosphorylation could inhibit β-catenin separation from the cell membrane, preventing it from entering the nucleus and performing its function and promoting the progression of cancer (44, 45). In addition, the downstream apoptosis process-related genes, such as Bcl-XL, Bcl-2, and BAD, were also affected and induced the apoptosis process, consistent with their functions (46). It has been shown that mortalin has an irreplaceable role in the regulation of protein phosphorylation and causes angiogenesis and sorafenib resistance in HCC.

CaA is a phenolic compound synthesized abundantly by plants that is found in the basic skeleton of many phytochemicals, including CAPE (47, 48). For the antioxidant activity, anti-atherosclerotic activity, antiproliferative activity, and other various pharmacologic actions of CaA and its derivatives, they have been widely employed in clinical treatment so far and were recognized as antithrombotic, antihypertensive, antidiabetic, anticancer, and anti-inflammatory agents (49, 50). In addition, it was reported that the expression level of mortalin showed significant correlation with HCC progression, and there was a high level of mortalin autoantibody in serum of patients with liver cirrhosis, suggesting that mortalin could be a promising serological marker for diagnosis (51). The relationship between mortalin, HCC, and CaA treatment remains to be further explored. Previously, we found that CaA may alter the progression of HCC through various means. To inhibit angiogenesis, CaA attenuated the activation of hypoxia-inducible factor (HIF)-1α by reducing c-Jun N-terminal kinase (JNK)1 activation and reducing HIF-1α stabilization and decreased p-STAT-3, attenuating the recruitment of HIF-1α and p-STAT-3 to the VEGF promoter (30). We also found that CaA attenuated cancer stem cell-like properties through the epigenetic regulation of the transforming growth factor (TGF)β–mothers against decapentaplegic homolog 2 (SMAD2) signaling pathway (31). In addition, CaA could block the expression/secretion of endogenous interleukin (IL)-6 by attenuation of the NF-κB–IL-6–STAT-3 feedback loop in HCC cells (28). Nevertheless, these effects induced by CaA are all dependent on an indirect means of signal transduction. However, our present study revealed that CaA also exhibited targeted intervention to mortalin by inducing the ubiquitin-mediated degradation of mortalin in HCC. Combined with the above evidence, we infer that the application of CaA may be a promising clinical targeted therapy for HCC in the future.

## Conclusions

**Figure 7:**
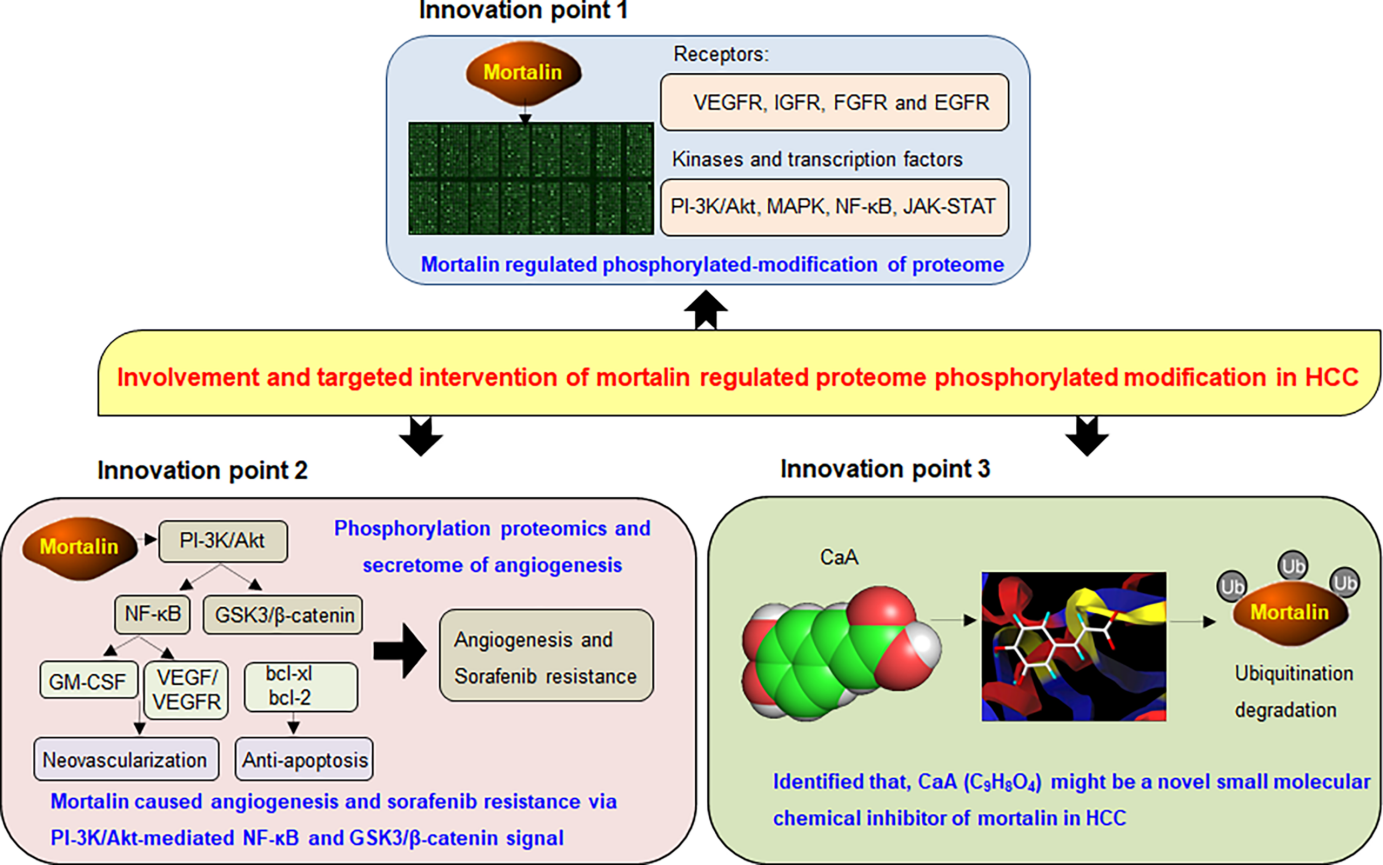
A sketch map summarizing the conclusions, innovations, and potential clinical significance of our present study.

Our present study revealed that mortalin, which regulated the phosphorylation of cancer-associated proteins and angiogenesis-related secretome, caused angiogenesis and sorafenib resistance in HCC. Among them, PI3K/Akt regulated NF-κB and β-catenin signaling, two neoplastic biological processes that play important roles in angiogenesis and sorafenib resistance of HCC. Moreover, mortalin was a competitive risk factor for HCC, predicting poor prognosis and the poor curative effect of sorafenib. Furthermore, we found that CaA was a novel chemical inhibitor targeting mortalin in HCC (**Figure 7**). Our present study not only reveals the key process of mortalin-induced HCC progression and the anticancer functions induced by CaA and other phytochemicals with a similar structure but also provides a new theoretical basis for further research into the targeted intervention of HCC.

## Data Availability Statement

The original contributions presented in the study are included in the article/**Supplementary Material**. Further inquiries can be directed to the corresponding authors.

## Ethics Statement

The studies involving human participants were reviewed and approved by Medical Ethics Committee of the Affiliated Changzhou No. 2 Hospital of Nanjing Medical University. The patients/participants provided their written informed consent to participate in this study. The animal study was reviewed and approved by Nanjing Medical University Institutional Animal Care and Use Committee.

## Author Contributions

LT and LL conceived the research. YY and MJ drafted the manuscript. MJ revised the manuscript carefully. YY, MJ, and WS performed cell experiments, phospho-specific protein microarray, angiogenic antibody array, and biological functional analysis. YD and SC carried out the *in vivo* xenograft and IHC/TUNEL. RC and HY carried out the patient’s sample preparation, follow-up, and statistical analysis of the data. All authors contributed to the article and approved the submitted version.

## Funding

This work was supported by the National Natural Science Foundation of China (81973096), the Natural Science Foundation of Jiangsu Province (BK20181155), and the Project Funded by Jiangsu Postgraduate Research and Innovation Plan (SJCX19-0316).

## Conflict of Interest

The authors declare that the research was conducted in the absence of any commercial or financial relationships that could be construed as a potential conflict of interest.

## Publisher’s Note

All claims expressed in this article are solely those of the authors and do not necessarily represent those of their affiliated organizations, or those of the publisher, the editors and the reviewers. Any product that may be evaluated in this article, or claim that may be made by its manufacturer, is not guaranteed or endorsed by the publisher.

## Abbreviations

AKT, protein kinase B (PKB); Bcl-XL, B-cell leukemia XL; GSK3β, glycogen synthase kinase 3β; HCC, hepatocellular carcinoma; NF-κB, nuclear factor κB; PI3K, phosphatidylinositol 3-kinase; ROS, reactive oxygen species; VEGFR2, vascular endothelial growth factor receptor 2; GM-CSF, granulocyte-macrophage colony-stimulating factor.
