# Supplementary material for: Involvement and Targeted Intervention of Mortalin-Regulated Proteome Phosphorylated-Modification in Hepatocellular Carcinoma
Source: Front Oncol. 2021 Jul 29;11:687871. doi: 10.3389/fonc.2021.687871 (PMC8358780; doi:10.3389/fonc.2021.687871)
Supplement: Supplementary file 1 [file DataSheet_1.docx]

**Involvement and Targeted Intervention of Mortalin-regulated Proteome Phosphorylated-modification in Hepatocellular Carcinoma**

**Ye Yang ^1†^, Ming Jin ^1†^, Yi Dai ^2^, Wenqi Shan ^1^, Shuai Chen ^2^, Rong Cai ^1^, Haojun Yang ^2^, Liming Tang ^2*^ , and Lei Li ^1*^**

Note: †These authors have contributed equally to this work and share first authorship.

^1^ Center for Global Health, School of Public Health, Nanjing Medical University, Nanjing, 211166, China.

^2^ Department of General Surgery, the Affiliated Changzhou No. 2 Hospital of Nanjing Medical University, Changzhou, 213003, China.

*** Correspondence:**

Dr. Liming Tang, Department of General Surgery, the Affiliated Changzhou No. 2 Hospital of Nanjing Medical University, Changzhou, 213003, China. Tel: +86-0519-8108-7666, Fax: +86-0519-8108-7711, E-mail: [liming_tang@sina.cn](mailto:liming_tang@sina.cn); Dr. Lei Li, Center for Global Health, School of Public Health, Nanjing Medical University, Nanjing, 211166, China. Tel: +86-25-8686-8, Fax: +86-25-8652-7613, E-mail: [lilei@njmu.edu.cn](mailto:lilei@njmu.edu.cn).

**SUPPLEMENTARY MATERIALS AND METHODS**

**Cell transfection**

Cells in logarithmic growth phase were collected to prepare cell suspension. The cells were seeded in 6-well plates at a density of 2×10^5^ per well for 24 h. PcDNA-3.1- mortalin-flag plasmids were synthesized by Generay Biotech Co. Ltd (Shanghai, China), while the negative control (NC)-siRNA and mortalin-siRNA were purchased from Santa Cruz Biotechnology, siRNAs used in this study were list in Supplementary Table S1. Briefly, 5 ng/ml of plasmids or 20 nM of siRNAs were mixed with lipofectamine 3000 reagent (Invitrogen, Carlsbad, USA) in mediums containing 10% FBS without antibiotics. After 12 h, the cells were cultured in fresh mediums supplemented with 10% FBS for another 24 h before being used for other experiments.

**Capillary tube formation assay**

As we described previously ([1](#_ENREF_1)), 1×10^4^ of HUVECs were seeded in a 24-well plate on matrigel that had polymerized for 30 min at 37 ˚C. After then, such cells were incubated in the mediums which were mixed in equal proportions of ECM mediums (Invitrogen, Carlsbad, CA, USA) and conditioned mediums that collected from NC-siRNA, mortalin-siRNA, or mortalin-plasmids transfected HCC cells for another 6 h, respectively. Capillary morphogenesis was evaluated by using an inverted microscope (Olympus, Tokyo, Japan), and quantitated in five randomly chosen fields.

**Cell viabilities and calculation of the 50% inhibitory concentrations (IC_50_)**

In 96-well plates, 2×10^3^ cells were seeded and treated as indicated for 24 h. Cell viability was determined by incubating the cells with 10.0 μL of CCK-8 solution (Beyotime) for 4 h. The absorbance was measured with a microplate reader (Bio-Rad, CA, USA) at a test wavelength of 450 nm, and the IC_50_ was calculated using a GraphPad 8.0 software (CA, USA). The cell viability was determined in triplicate. Through a three-parameter dose-response equation and nonlinear regression, the inhibition rates were calculated and sigmoidal curves were generated. The ordinate represents the inhibition rate and the abscissa represents the concentration (log); the results were displayed as “best fit values” ± “standard errors” ([2](#_ENREF_2)).

**Western blot**

Total protein was extracted by lysing cells in RIPA buffer (Beyotime), then the concentrations were measured with the BCA kit (Beyotime). Afterwards, proteins were separated by 10% sodium dodecyl sulfate-polyacrylamide gel electrophoresis followed by transferring to polyvinylidene fluoride membranes (Millipore, Billerica, USA)([3](#_ENREF_3)). After blocking with 10% non-fat milk in TBST , membranes were incubated with the primary and secondary antibodies. Antibodies used were listed in Supplementary Table S5. The immune complexes were detected by an enhanced chemiluminescence kit (Cell Signaling Technology).

**Enzyme-linked immunosorbent assay (ELISA)**

To analyze VEGF and GM-CSF secretion, we performed ELISA using the human VEGF Quantikine kit and Human GM-CSF Quantikine Kit (R&D Systems, MN, USA). Briefly, 0.5 μg/mL VEGF antibody was added to each well of a 96-well polyvinyl microplate (R&D Systems) and stored at 4ºC overnight. Sample (50 μL) or standard protein (Recombinant human VEGF, R&D Systems) was added to the wells. After incubation, the plates were washed with phosphate buffered saline (PBS); 50 ng/mL biotinylated VEGF antibody (R&D Systems) was added prior to incubation with streptavidin-HRP. For the colorimetric reaction, 2,2'-azinobis (3-ethylbenzothiazoline-6-sulfonicacid)-diammonium salt (ABTS, Sigma) was added, and the absorbance of the wells was measured at 450 nm using a multi-well plate reader (Model 680, Bio-Rad, USA). For GM-CSF, add 100 μl of standard, control, or sample per well. Incubate for 2 hours at room temperature. Aspirate each well and wash with Wash Buffer (400 μl) , repeating the process three times for a total of four washes. remove any remaining Wash Buffer by aspirating or decanting. Invert the plate and blot it against clean paper towels. Add 200 μl of Human GM-CSF Conjugate to each well and cover with a new adhesive strip. Samples incubated for 1-2 h at room temperature. Repeat the aspiration/wash step. Add 200 μl of Substrate Solution to each well. Incubate for 20 minutes at room temperature and protect from light. Add 50 μl of Stop Solution to each well. Determine the optical density of each well within 30 minutes, using a microplate reader set to 450 nm.

**Determination of cell apoptosis by flow cytometry**

Treated cells were harvested with trypsin-EDTA, washed with PBS by centrifugation and fixed with 1 ml of ice-cold 70% ethanol overnight. As we described previously ([1](#_ENREF_1)), the fixed cells were centrifuged, suspended in lysis buffer and incubated with RNase A for 10 min at room temperature. Cell apoptosis analyses were performed using Annexin V-FITC and propidium iodide (PI) kit (Beyotime) according to the manufacturer’s instruction, followed by flow cytometry analysis.

**SUPPLEMENTARY Tables**

**Table. S1. siRNAs used in this study**

| siRNAs | Web Link |
| --- | --- |
| Mortalin | https://datasheets.scbt.com/sc-35520.pdf |
| NC | <http://datasheets.scbt.com/sc-37007.pdf> |

**Table. S2. The criteria of immunostaining and TUNEL staining score**

| Staining | Criteria of score * | Range of score |
| --- | --- | --- |
| Mortalin | intensity or intensity + heterogeneity | 0-3 or 0-7 |
| TUNEL | TUNEL-positive cells |  |
| CD34 | intensity | 0-3 |

The immunostaining and TUNEL staining scores were semi-quantified by Quick-score (Q-score) based on intensity and heterogeneity (positive rates) ([1](#_ENREF_1)). The score of the staining intensity was presented as 0 point (none), 0-1 point (low), 1-2 points (medium), and 2-3 points (high). The positive rates were scored as 0 point (0%), 1 point (1-25%), 2 points (26-50%), 3 points (51-75%), and 4 points (76-100%). The Q-score was the sum of heterogeneity and intensity.

**Note:** * In our present study, the positive rates of mortalin are high in HCC tissue samples, so we only used the intensity as the criteria of score, and the expression of mortalin was defined as high when the scores were ≥ 2. In Huh7^SR^ xenografts, we used the sum of heterogeneity and intensity as the criteria of score. The data for TUNEL-positive cells were provided as the mean of each experimental group calculated in 10 high-power fields. The immunostaining of CD34 was scored by intensity and the expression was defined as positive when the score ≥ 1. The inclusion criteria of intratumoral microvessels was dependent on the CD34 positive endothelial cells according to the previous study ([4](#_ENREF_4)). The number of microvessels was provided as the mean of each experimental group calculated in 10 high-power fields.

**Table. S3. Clinicopathological correlation of Mortalin in HCCs**

| Characteristic | Number of cases | | χ^2^ | *p* value * |
| --- | --- | --- | --- | --- |
|  | Mortalin-Low | Mortalin-High |  |  |
| Age (years) |  |  |  |  |
| ≤ 50 | 28 | 37 | 0.258 | 0.611 |
| > 50 | 30 | 31 |  |  |
| Gender |  |  |  |  |
| Male | 50 | 59 | 0.029 | 0.865 |
| Female | 8 | 9 |  |  |
| HBsAg |  |  |  |  |
| Negative | 5 | 7 | 0.0002 | 0.989 |
| Positive | 53 | 61 |  |  |
| Hepatic fibrosis |  |  |  |  |
| S1/S2 | 29 | 40 | 0.659 | 0.417 |
| S3/S4 | 29 | 28 |  |  |
| Serum AFP |  |  |  |  |
| ≤ 20 ng/ml | 18 | 8 | 5.969 | 0.015 |
| > 20 ng/ml | 40 | 60 |  |  |
| Tumor size |  |  |  |  |
| ≤ 5 cm | 32 | 21 | 6.614 | 0.01 |
| > 5 cm | 26 | 47 |  |  |
| Multinodular tumor |  |  |  |  |
| No | 45 | 54 | 0.0009 | 0.975 |
| Yes | 13 | 14 |  |  |
| TNM stage |  |  |  |  |
| I/II | 31 | 11 | 17.926 | < 0.0001 |
| III/IV | 27 | 57 |  |  |

**Table. S4. Clinicopathological correlation of Mortalin in recurrent HCCs**

| Characteristic | Number of cases | | *p* value ** |
| --- | --- | --- | --- |
|  | Mortalin-Low | Mortalin-High |  |
| Age (years) |  |  |  |
| ≤ 50 | 4 | 16 | 0.531 |
| > 50 | 5 | 9 |  |
| Gender |  |  |  |
| Male | 8 | 23 | 0.687 |
| Female | 1 | 2 |  |
| HBsAg |  |  |  |
| Negative | 2 | 3 | 0.846 |
| Positive | 7 | 22 |  |
| Hepatic fibrosis |  |  |  |
| S1/S2 | 3 | 4 | 0.534 |
| S3/S4 | 6 | 21 |  |
| Serum AFP |  |  |  |
| ≤ 20 ng/ml | 2 | 0 | 0.109 |
| > 20 ng/ml | 7 | 25 |  |
| Tumor size |  |  |  |
| ≤ 5 cm | 6 | 5 | 0.032 |
| > 5 cm | 3 | 20 |  |
| Multinodular tumor |  |  |  |
| No | 7 | 18 | 0.917 |
| Yes | 2 | 7 |  |
| TNM stage |  |  |  |
| I/II | 1 | 0 | 0.588 |
| III/IV | 8 | 25 |  |

The patients listed in Table. S1 underwent curative liver resection for primary tumors between Jan 2013 and Dec 2017 at the Affiliated Changzhou No. 2 Hospital of Nanjing Medical University. The inclusion criteria of the patient cohort included (1) having a distinctive pathologic diagnosis of HCC and (2) surgical resection, defined as complete resection of all tumor nodules with the cut margin being free of cancer by histologic examination. The histological type and grade were reviewed by two individual board-certified pathologists. The exclusion criterion of patients was having prior anticancer treatment before liver resection. For the collection of follow-up data, all the 126 patients were followed after surgical treatment until Dec 2019. During the follow-up, patients were monitored every 3 months. The computed tomography scanning (CT) and/or magnetic resonance imaging (MRI) were performed when tumor recurrence was suspected. The 34 advanced recurrent HCC patients’ tissue specimens and clinical data were as we described previously ([1](#_ENREF_1)). They were received combined sorafenib treatment and transarterial chemoembolization therapy (TACE, when recurrence), after the curative liver resection. The inclusion and/or exclusion criteria, the review of histological type and grade, and the collection of follow-up data were the same as described above.

**Note:** * The analyses were conducted using the corrected χ^2^ tests, and ** the analyses were conducted using the Fisher’s Exact Test.

**Table S5. Antibodies used in this study**

| Antibodies | Web Link | Used |
| --- | --- | --- |
| Mortalin | https://www.cst-c.com.cn/datasheet.jsp?productId=3593&images=1&protocol=0 | 1: 1000 (WB)  1: 100 (IHC) |
| p-PI3K | https://www.cellsignal.cn/datasheet.jsp?productId=17366&images=1&protocol=0 | 1: 1000 |
| p-Akt | https://www.cst-c.com.cn/datasheet.jsp?productId=4060&images=1&protocol=0 | 1: 2000 |
| p-NF-κB p65 | https://www.cellsignal.cn/datasheet.jsp?productId=3031&images=1&protocol=0 | 1: 1000 |
| p-GSK3α/β | https://www.abcam.cn/gsk3-alpha--beta-phospho-y216--y279-antibody-ab4797.html | 1: 1000 |
| p-βCatenin | https://www.cellsignal.cn/datasheet.jsp?productId=9565&images=1&protocol=0 | 1: 1000 |
| p-VEGFR2 | https://www.cellsignal.cn/datasheet.jsp?productId=2471&images=1&protocol=0 | 1: 1000 |
| Bcl-XL | https://www.cellsignal.cn/datasheet.jsp?productId=2764&images=1&protocol=0 | 1: 1000 |
| ubiquitin | https://www.cst-c.com.cn/datasheet.jsp?productId=3933&images=1&protocol=0 | 1: 500 |
| Flag | https://www.beyotime.com/Manual/AF519%20Flag%E6%8A%97%E4%BD%93.pdf | 1: 1000 |
| β-Actin | https://www.beyotime.com/Manual/AA128%20Actin%E6%8A%97%E4%BD%93.pdf | 1: 1000 |

**SUPPLEMENTARY FIGURES and their Legends**

**Fig. S1. Transfection efficiency**


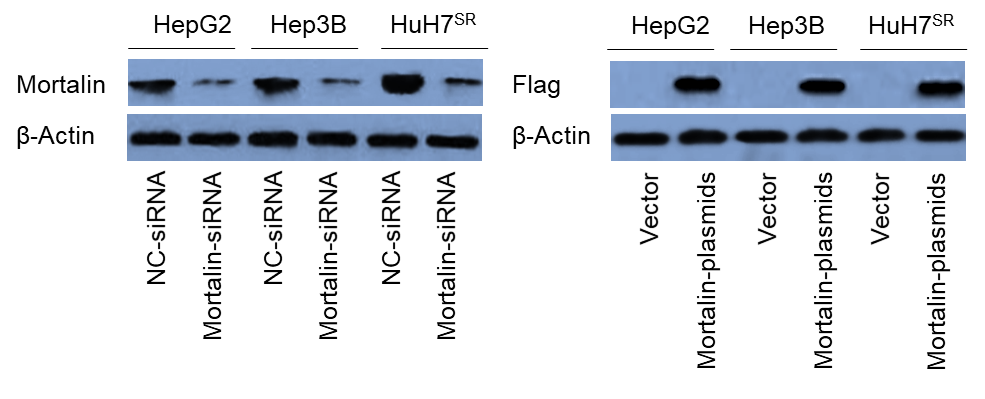


HCC cells were transfected by mortalin-siRNA or mortalin-plasmids. Western blot analyses of the expressions of mortalin or Flag.

**Fig. S2. Fifty altered neighbour factors around mortalin**


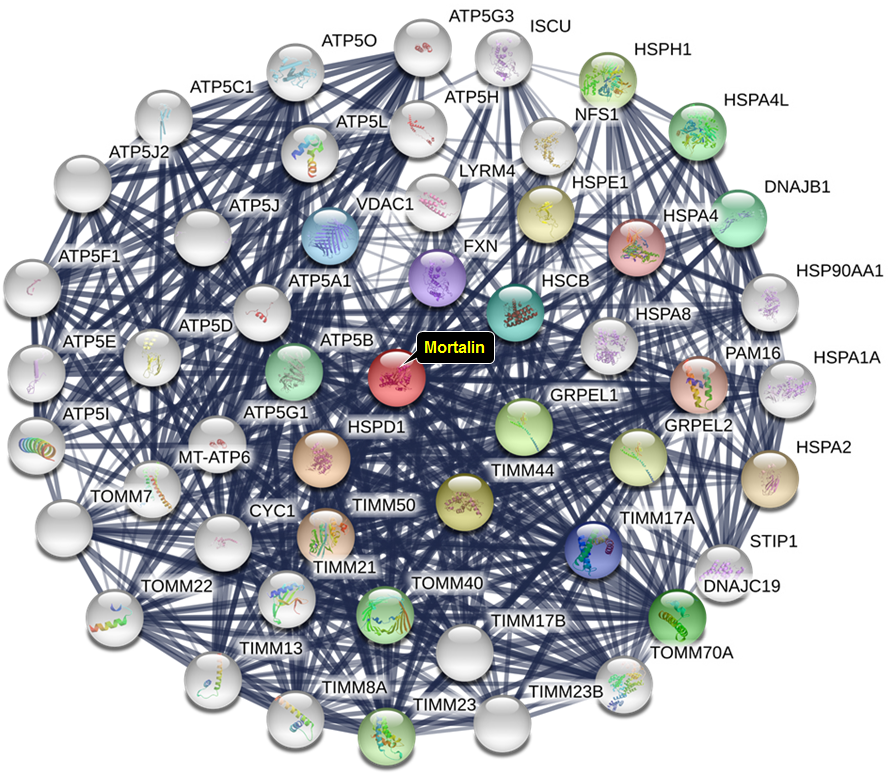


The protein-protein interaction with the 20 most frequently altered neighbor interactors and 30 indirect interactors around mortalin generated by STRING.

**Fig. S3. KEGG enrichment analysis of phospho-antibody microarray**


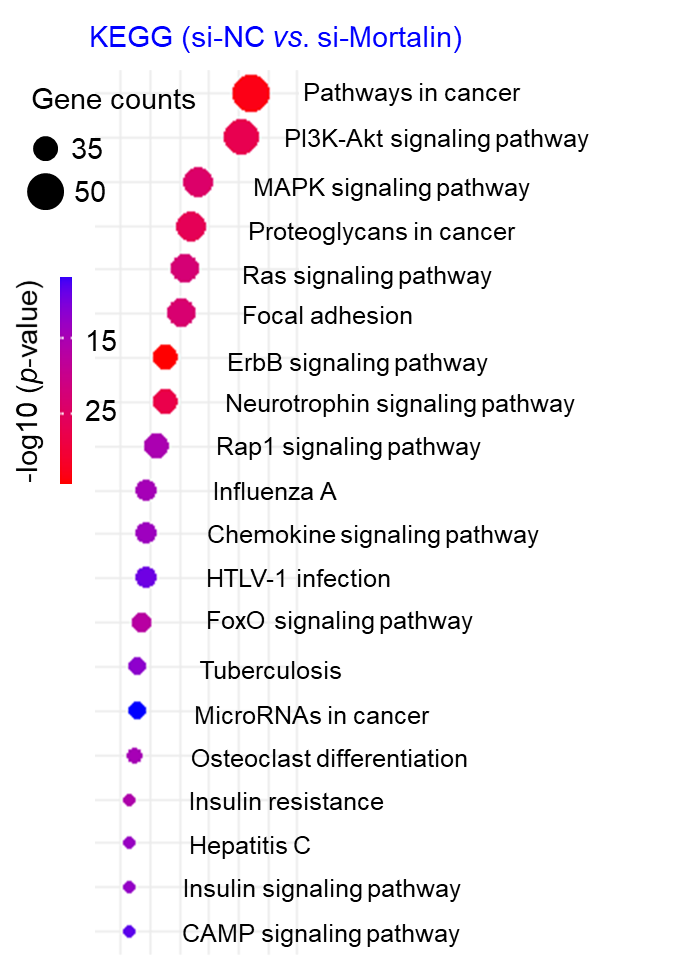


The top 20 pathways of the proteins whose phosphorylation levels upregulated/ downregulated more than 1.5-fold upon mortalin-knockdown.

**Fig. S4. Confirm the effects of CaA *in vivo***

**
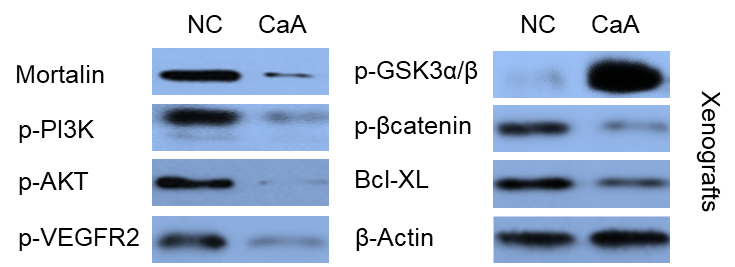
**

Western blotting analyses of the expression of the proteins in a previously established xenograft model of MHCC97H cells, which was treated by CaA.

**SUPPLEMENTARY References**

1. Shen J, Jiang F, Yang Y, Huang G, Pu F, Liu Q, et al. 14-3-3eta is a novel growth-promoting and angiogenic factor in hepatocellular carcinoma. *J Hepatol* (2016) 65(5):953-62. Epub 2016/10/19. doi: 10.1016/j.jhep.2016.05.017. PubMed PMID: 27210426.

2. Qiu Y, Dai Y, Zhang C, Yang Y, Jin M, Shan W, et al. Arsenic trioxide reverses the chemoresistance in hepatocellular carcinoma: a targeted intervention of 14-3-3eta/NF-kappaB feedback loop. *J Exp Clin Cancer Res* (2018) 37(1):321. Epub 2018/12/24. doi: 10.1186/s13046-018-1005-y. PubMed PMID: 30572915; PubMed Central PMCID: PMCPMC6302299.

3. Jiao K, Zhen J, Wu M, Teng M, Yang K, Zhou Q, et al. 27-Hydroxycholesterol-induced EndMT acts via STAT3 signaling to promote breast cancer cell migration by altering the tumor microenvironment. *Cancer biology & medicine* (2020) 17(1):88-100. doi: 10.20892/j.issn.2095-3941.2019.0262. PubMed PMID: 32296578; PubMed Central PMCID: PMC7142833.

4. Weidner N. Current pathologic methods for measuring intratumoral microvessel density within breast carcinoma and other solid tumors. *Breast cancer research and treatment* (1995) 36(2):169-80. PubMed PMID: 8534865.
